# The complete chloroplast genome sequence of *Chimonanthus praecox* cv. *concolor*

**DOI:** 10.1080/23802359.2019.1669084

**Published:** 2019-09-24

**Authors:** Yujie Zhao, Yuan Ren, Yunfang Xu, Ming Yan, Yan Huo, Xueqing Zhao, Zhaohe Yuan

**Affiliations:** a Co-Innovation Center for Sustainable Forestry in Southern China, College of Forestry, Nanjing Forestry University, Nanjing, China;; b College of Forestry, Nanjing Forestry University, Nanjing, Jiangsu, China

**Keywords:** *Chimonanthus praecox* cv. *concolor*, chloroplast genome, phylogeny analysis

## Abstract

Calycanthaceae is a perennial shrub endemic to China with important ornamental and medicinal values. In this study, *Chimonanthus praecox* cv. *concolor* chloroplast (cp) genome was characterized using Illumina paired-end reads data. In total, whole cp genome is 153,254 bp long and contains a small single-copy region of 19,769 bp, a pair of repeat (IRa and IRb) regions of 23,286 bp each, and a large single-copy region of 86,913 bp. This genome contains 129 genes, including 84 protein-genes, 8 rRNA genes, and 37 tRNA genes. Phylogenetic analysis based on 19 cp genomes showed that *C. praecox* cv. *concolor* is closely related to *Chimonanthus praecox* and *Chimonanthus nitens*.

Calycanthaceae, widely cultivated in many provinces in China, is a Chinese endemic winter-flowering plant which has a long history of cultivation. The chloroplast (cp) DNA has been widely and successfully employed by many plant biologists for the reconstruction of plant evolution (Kellogg 1998, Goremykin et al. 2003). Good knowledge of complete cp genomics is helpful to the establishments of effective management and conservation strategies for species. Studies about Calycanthaceae such as classification, ecological character (Liu et al. 1996) and genetic diversity (Zhou and Ye 2002) have been done. However, genomic information of *C. praecox* cv. *concolor* is less well known. Here, in this study, we report the complete cp genome of *C. praecox* cv. *concolor* so that the data can provide evidence for Calycanthaceae genomics and phylogenetic research.

Total DNA (Voucher specimen: E118°49′1.19″, N32°4′47.24″, CF201903079, NFU) was stored in the forest breeding laboratory of Nanjing Forestry University. It was extracted from leaf tissue from *C. praecox* cv. *concolor*. After filtered and trimmed by fastp (Chen et al. 2018), high-quality paired-end reads were assembled into a complete cp genome using GetOrganelle (Jin et al. 2018) using *Calycanthus floridus* var. *glaucus* (NC_004993.1) as reference. The genome annotation was performed with GeSeq (Tillich et al. 2017) and by aligning with the cp genome of relatively related species within the Calycanthaceae, including *C. floridus* var. *glaucus* and *Calycanthus chinensis* (NC_037504.1). The annotation result was inspected using Geneious and adjusted manually as needed. Finally, a physical map of the genome was drawn by using the IQ-Tree program (Nguyen et al., 2015). The annotated cp genome has been submitted to GenBank under the accession number MN172352.

The cp genome was 153,254 bp in size with a typical quadripartite structure, consisting of a large single-copy (LSC, 86,913 bp), a small single-copy (SSC, 19,769 bp), and two inverted repeats (IRs, 23,286 bp each). In total, this cp genome contained 129 genes, including 84 protein-coding genes, 8 rRNA genes, and 37 tRNA genes. Most of the genes occurred as single copy in the LSC or SSC, but 15 genes presented as two copies in the IRs, including all 4 rRNA genes (*rrn 4.5*, *rrn 5*, *rrn 16*, and *rrn 23*), 7 tRNA genes (*trnI-CAU*, *trnL-CAA*, *trnV-GAC*, *trnI-GAU*, *trnA-UGC*, *trnR-ACG*, and *trnN-GUU*), and 4 protein-coding genes (*rpl23*, *ycf2*, *ndhB*, and *rps7*). Among all the genes, 13 genes (*rps16*, *atpF*, *rpoC1*, *nahB*, *ndhA*, *rpl2*, *rpl16*, *petB*, *petD*, *trnL-UAA*, *trnV-UAC*, *trnI-GAU*, and *trnA-UGC*) contained a single intron, but 3 genes (*ycf3*, *rps12*, *clpP*) contained two introns.

Based on 19 cp genome sequences, the phylogenetic position of *C. praecox* cv. *concolor* was inferred using the maximum-likelihood (ML) method. The alignment was achieved by MAFFT (Katoh and Standley 2013), and the phylogenetic tree was constructed using IQ-tree. The best-fitted model was selected by ModelFinder (Kalyaanamoorthy et al. 2017). The result (Figure 1) indicated that *C. praecox* cv. *concolor* was closely related to *Chimonanthus praecox* and *Chimonanthus nitens*. We believe that the complete cp genome of *C. praecox* cv. *concolor* could be subsequently utilized for classification purpose.

**Figure 1.**
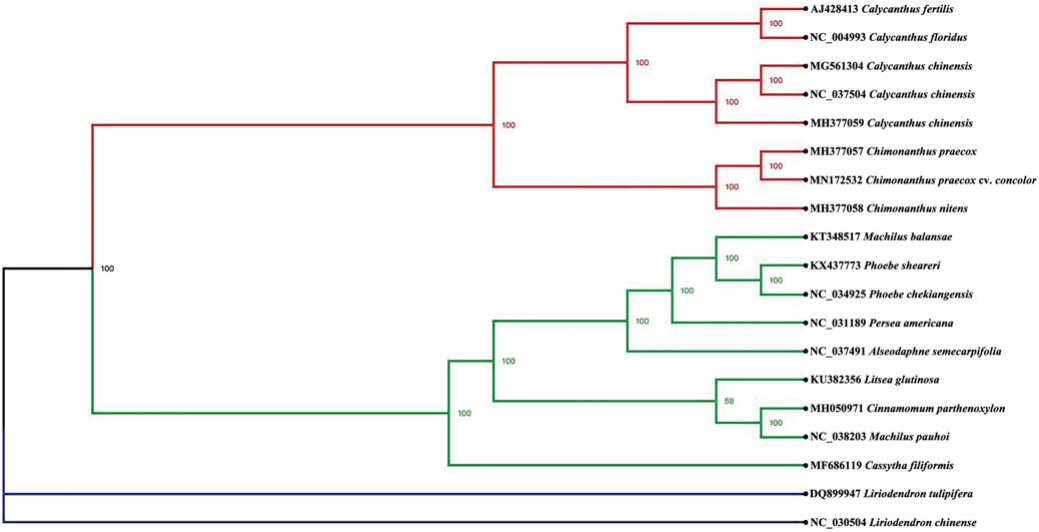
Phylogenetic position of *Chimonanthu praecox* cv. *concolor* within Laurales. Phylogenetic tree was constructed using ML method. Numbers in the nodes were the support values.
